# Cortical and Subcortical Grey and White Matter Atrophy in Myotonic Dystrophies Type 1 and 2 Is Associated with Cognitive Impairment, Depression and Daytime Sleepiness

**DOI:** 10.1371/journal.pone.0130352

**Published:** 2015-06-26

**Authors:** Christiane Schneider-Gold, Barabara Bellenberg, Christian Prehn, Christos Krogias, Ruth Schneider, Jan Klein, Ralf Gold, Carsten Lukas

**Affiliations:** 1 Department of Neurology, St. Josef-Hospital, Ruhr-University Bochum, Bochum, Germany; 2 Department of Radiology, St. Josef-Hospital, Ruhr-University Bochum, Bochum, Germany; 3 Fraunhofer-MEVIS, Institute for Medical Image Computing, Bremen, Germany; University of Texas MD Anderson Cancer Center, UNITED STATES

## Abstract

**Objectives:**

Central nervous system involvement is one important clinical aspect of myotonic dystrophy type 1 and 2 (DM1 and DM2). We assessed CNS involvement DM1 and DM2 by 3T MRI and correlated clinical and neuocognitive symptoms with brain volumetry and voxel-based morphometry (VBM).

**Methods:**

12 patients with juvenile or classical DM1 and 16 adult DM2 patients underwent 3T MRI, a thorough neurological and neuropsychological examination and scoring of depression and daytime sleepiness. Volumes of brain, ventricles, cerebellum, brainstem, cervical cord, lesion load and VBM results of the patient groups were compared to 33 matched healthy subjects.

**Results:**

Clinical symptoms were depression (more pronounced in DM2), excessive daytime sleepiness (more pronounced in DM1), reduced attention and flexibility of thinking, and deficits of short-term memory and visuo-spatial abilities in both patient groups. Both groups showed ventricular enlargement and supratentorial GM and WM atrophy, with prevalence for more GM atrophy and involvement of the motor system in DM1 and more WM reduction and affection of limbic structures in DM2. White matter was reduced in DM1 in the splenium of the corpus callosum and in left-hemispheric WM adjacent to the pre- and post-central gyrus. In DM2, the bilateral cingulate gyrus and subgyral medio-frontal and primary somato-sensory WM was affected.

Significant structural-functional correlations of morphological MRI findings (global volumetry and VBM) with clinical findings were found for reduced flexibility of thinking and atrophy of the left secondary visual cortex in DM1 and of distinct subcortical brain structures in DM2. In DM2, depression was associated with brainstem atrophy, Daytime sleepiness correlated with volume decrease in the middle cerebellar peduncles, pons/midbrain and the right medio-frontal cortex.

**Conclusion:**

GM and WM atrophy was significant in DM1 and DM2. Specific functional-structural associations related morphological changes to cognitive impairment, depression and daytime sleepiness, partly indicating involvement of complex neuronal networks.

## Introduction

Myotonic dystrophy type 1 and 2 (DM1 and DM2) are clinically similar yet distinct autosomal dominantly inherited multi-systemic diseases associated with two different polyglutamine repeat expansion mutations [1, 2]. Despite evidence for similar pathophysiological downstream effects of the mutations [3], cognitive impairment and brain magnetic resonance imaging (MRI) findings (white matter (WM) and grey matter (GM) pathology) were shown to be different in both diseases with more pronounced clinical and MRI abnormalities in DM1 in most studies [4, 5]

Previous MRI based studies quantified WM affection either by morphometric techniques (brain volumetry or voxel-based-morphometry (VBM)) or assessed nerve fiber integrity using diffusion-tensor-imaging (DTI) and tractography. Brain WM was described to be preferentially involved in DM1 and DM2 [4, 6–10]. In a recent VBM study by Minnerop et al., extensive WM atrophy of all cortical lobes, the corpus callosum and the brainstem was detected in DM1 and DM2 [8]. For DM1, signs of microstructural WM damage in interhemispheric, corticospinal, and limbic pathways and in frontal, temporal, parietal and occipital WM were confirmed by DTI studies [8–13].

GM involvement by cortical and subcortical atrophy has been reported in different studies mostly regarding DM1, but also for the DM2 subtype. In DM1, bilateral GM atrophy of the frontal-, temporal- and parietal cortex and the superior occipital gyrus was described [5, 8, 11, 14], but also deep GM atrophy of the thalamus, putamen and caudate nuclei was detected [5, 8, 11]. Controversial results have been published regarding GM atrophy in DM2; while one study reported no cortical or subcortical GM involvement [8], others found frontal, parietal and occipital cortical atrophy, as well as thalamic and hippocampal GM atrophy [5, 7].

Other imaging modalities like molecular imaging ((single-photon emission computed tomography (SPECT) and positron emission tomography (PET)) and MR-spectroscopy (MRS) revealed reduced blood flow and glucose utilisation of frontal areas with more widespread hypoperfusion in DM1 than in DM2 [15], hypometabolism of the left frontal lobe in DM1 [16], bilateral frontotemporal hypometabolism in DM1 and to a lesser extent in DM2 [5], and neurometabolic alterations of GM and WM in DM1 and DM2 [17].

Involvement of infratentorial structures has been found in both DM subtypes, reflected by atrophy of the brainstem and the cerebellar cortex [5, 7, 8]. In a recent study using transcranial ultrasound, significant association between excessive daytime sleepiness and the echogeneity of the brainstem Raphe was detected exclusively in DM1, suggesting a specific functional-structural relevance of brainstem structures in DM1 [18].

Although considerable structural CNS involvement on one hand and cognitive deficits on the other hand have equivocally been detected in the myotonic dystrophies (DMs), only few studies have found significant correlations between these features [5, 11, 13], while others found none [8, 16].

Referring to those previous studies, we hypothesize that there is widespread and heterogeneous central nervous system (CNS) involvement in the DMs and that distinct structural MRI changes may correspond to CNS symptoms in DM1 and DM2. We therefore include comparable groups of DM1 and DM2 patients.

As suggested by a recent neuropsychological study on DM1 [19], we hypothesize that in DMs dysfunction may occur not only in distinct CNS regions but also in complex neuronal networks. We hypothesize that excessive daytime sleepiness may be related not only to brainstem neuronal cell degeneration or dysfunction [8, 18, 20] but to more widespread affection of sleep regulation structures.

## Materials and Methods

This study was approved by the local institutional regulatory board of the Medical Faculty of the Ruhr-University Bochum (registration number 3794–10) and written informed consent according to the Declaration of Helsinki was obtained from all individuals.

### Study population

A summary of the study population is given in Table 1. In total 12 DM1 patients with juvenile or classical and 16 adult DM2 patients with diseases onset after age of 16 years were recruited prospectively on an outpatient basis. One DM2 patient did not receive MRI. Exclusion criteria were: age at onset younger than 14 years, a history of stroke or other CNS disorders, sleep-apnoea syndrome or cancer. Patients with disease onset > 20 years were defined as adult and patients with disease onset between 14 and 20 years as juvenile. A control group consisted of 33 age and gender matched healthy individuals, without a history of neurological disease or drug abuse. This control group was used only for MRI based volume quantification. In contrast, for neuropsychological testing, we referred to the normative data provided by the statistical manuals of the tests.

**Table 1:** Demography and clinical status of the study population.

		DM1 (n = 12; m/f: 8/4)	DM2 (n = 16; m/f: 5/11)	controls (n = 33; m/f: 14/19)
**age (years)**	Mean ± SD	45 ± 13	52 ± 7	42 ± 14
	[median;range]	[48; 25–60]	[52, 39–65]	[41; 20–65]
**age at disease onset (years)**	Mean ± SD	29 ± 10	38 ± 7	-
	[median;range]	[22; 14–44]	[39; 24–49]	-
**Disease duration (years)**	Mean ± SD	18 ± 7	14 ± 9	-
	[median;range]	[20; 5–26]	[12; 7–41]	-
**CTG repeat expansions**	[median;range]	[450; 75–720]	-	-
**MIRS** ^a^	Mean ± SD	3.3 ± 1.2	2.5 ± 0.5	-
	[median;range]	[4; 1–4]	[2; 2–3]	-
	P-value^d^	*P = 0*.026*		-
**ESS** ^b^	Mean ± SD	11.0 ± 4.8	7.4 ± 5.8	-
	[median;range]	[11.0; 5–20]	[6.5; 0–20]	-
	P-value^d^	*P = 0*.*109*		-
	Path.:non-path.^e^	5:7 [58%]	12:4 [25%]	-
**BDI** ^c^	Mean ± SD	7.6 ± 7.5	15.1 ± 12.5	-
	[median;range]	[5.0; 0–27]	[11.5; 1–42]	-
	P-value^d^	*P = 0*.*165*		-
	Path.:non-path.^e^	9:3 [25%]	8:8 [50%]	-

^a^: MIRS = muscular impairment rating scale;

^b^: ESS = Epworth daytime sleepiness score,

^c^: BDI = Beck’s depression inventory score.

^d^: P = statistical significance of group differences by Mann-Whitney-U tests (*: significant with P<0.050): DM1 compared to DM2.

^e^: Number of non-pathological: number of pathological cases [percentage of pathological cases]; cut-off values: Epworth daytime sleepiness score >10, Beck’s depression inventory > 11.

The DM1 or DM2 mutation was identified by DNA analysis of peripheral blood lymphocytes as described [21]. The number of CTG triplet repeats in the DM1 patients was retrieved from the genetic data. Since in DM2 the length of the CCTG-repeat expansion is not correlated with the severity of the disease, exact lengths of the repeat expansions were not determined. Details of the patients’ demography and the clinical status are provided in Table 2.

**Table 2:** Clinical details of the Myotonic Dystrophy (DM) patients.

N	Sex	group	age /years	Disease duration /years	age at onset /years	CTG expansions (blood)	MIRS Score^a^	Epworth sleepiness score	Restless legs syndrome	CNS symptoms
**1**	M	DM1	47	14	33	300	2	11		rigor of the left wrist
**2**	M	DM1	45	20	25	350	4	0		
**3**	F	DM1	59	23	36	600	4	11		
**4**	M	DM1	42	23	19	700	4	8		
**5**	M	DM1	54	20	34	500	4	15		rigor of the left wrist
**6**	F	DM1	50	8	42	75	1	12		
**7**	F	DM1	60	26	34	350	4	12		
**8**	M	DM1	25	10	15	750	3	5		Dysmetria of the left hand
**9**	F	DM1	26	12	14	750	3	18		
**10**	M	DM1	54	24	30	350	4	20		
**11**	M	DM1	26	5	21	750	2	7		
**12**	M	DM1	56	12	44	170	4	8		
**13**	F	DM2	53	16	37		3	0		sudden falls
**14**	M	DM2	47	7	40		3	20		
**15**	F	DM2	52	13	39		3	9	+	rigidness of the left wrist
**16**	F	DM2	47	8	39		3	13		dysmetria and tremor of the left hand
**17**	F	DM2	57				2	4		
**18**	F	DM2	57	8	49		2	0		
**19**	F	DM2	58	14	44		3	15		sudden falls, mild gait unsteadiness
**20**	F	DM2	46	8	38		3	7		
**21**	M	DM2	54	12	42		2	8		
**22**	F	DM2	59	10	49		3	9		mild gait unsteadiness
**23**	F	DM2	47	8	39		2	5	+	dysdiadocho-kinesia, of the left hand
**24**	M	DM2	59	30	29		2	6		dysmetria,of the left hand
**25**	F	DM2						0		
**26**	F	DM2	65	41	24		3	3	+	
**27**	M	DM2	43	13	30		2	6	+	
**28**	M	DM2	39	11	28		2	14		

^a^MIRS muscular impairment rating scale for DM1; adapted MIRS for DM2.

### Magnetic resonance imaging

Patients and healthy controls underwent MRI at 3 Tesla in a single centre. A 32-channel phased-array head coil was used in a standardized MRI protocol including:

1. isotropic T1- weighted 3D sequence for volumetry (3D T1 fast field echo, 180 sagittal slices, field of view: 240 mm, resolution: (1x1x1)mm^3^, repetition time, echo time, inversion time /ms: 10 / 4,6 / 1000, flip angle: 8°, turbo factor: 164; acquisition time: 6 min.),
2. isotropic 3D fluid attenuated inversion recovery (FLAIR) sequence for lesion quantification (3D FLAIR, 170 sagittal slices, field of view: 240 mm, resolution: (1x1x1)mm^3^, repetition time, echo time, inversion time /ms: 4800 / 286/ 1650, turbo factor: 182, acquisition time: 6’30” min.).

### Image analysis

#### Voxel-based morphometry (VBM) and Statistical Parametric Mapping (SPM)

Prior to VBM all 3D T1-weighted image series were corrected for WM lesions by lesion filling using the lesion segmentation toolbox for SPM8 [22]. Image processing was carried out using the VBM8 toolbox (http://dbm.neuro.uni-jena.de/vbm), based on statistical parametric mapping 8 (SPM8) (http://www.fil.ion.ucl.ac.uk/spm/) and MATLAB 8.0 with default settings for tissue segmentation of WM, GM and CSF and creation of probability maps in the MNI space. The series were bias corrected, tissue classified for GM and WM and registered using linear and non-linear transformations [23]. Subsequent analyses were performed on the GM and WM probability maps, which were multiplied by the non-linear components derived from the normalization matrix (‘non-linear modulation only’) to obtain relative GM and WM volumes corrected for differences in brain size. For smoothing an 8x8x8 mm^3^ Gaussian kernel was chosen. Absolute threshold masking with a threshold of 0.20 was used to avoid edge effects on the border between different tissue classes.

Voxelwise statistics used multiple regression analyses in SPM including age, subgroup type or clinical / neuropsychological / MRI parameters as covariates. In the regression analysis the covariates were incorporated under inclusion of an intercept and without centring. The resulting T-maps were explored after family-wise error (FWE) correction for multiple comparisons at cluster level. Cluster locations were presented in MNI-coordinates using neurological convention.

#### Volumetry post-processing

All volumes were derived from the individual T1-weighted 3D MRI datasets by an experienced reader using previously described semi-automated methods [24–29] yielding volumes of brain WM, GM, and intracranial cavity, cerebellum, brainstem and upper cervical cord (between the C1 and C3 vertebral level). GM and WM results were corrected for bias by white matter lesions by addition (WM) and subtraction (GM) of the total FLAIR lesion volume for each patient. Based on the lesion corrected GM and WM, as well as cerebellum and brainstem volumes the supratentorial brain volume was calculated.

Ventricular volumetry yielded the lateral ventricular volume (LVV), 3^rd^ ventricular volume (3VV), and 4^th^ ventricular volume (4VV). The volume of the temporal horns as part of the lateral ventricles was segmented in a separate procedure using unique geometrical landmarks to define the posterior limit of the temporal horns. The temporal horn index (THI) was calculated: THI = temporal horn volume / lateral ventricle volume and represents the relative widening of the temporal part of the lateral ventricles which has been proposed as an indirect and sensitive regional measure for hippocampal and parahippocampal atrophy [30].

The intracranial cavity volume (ICCV) was used for further normalization of all CNS volumes with respect to head size differences by multiplication of the results by the ratio of the mean ICCV of the whole cohort and the individual ICCV [31].

#### Corrections for physiological aging

Dependence of the CNS volumes on natural aging was checked in the healthy control group by a linear regression analysis and detected in the volumes of GM, supratentorial brain, lateral ventricle, 3^**rd**^. ventricle and the cerebellar volume. In those parameters the linear regression function of the control group was used to correct for the physiological age effects in all subjects.

#### Grading of white matter hyperintensities and Virchow-Robin-spaces

FLAIR hyperintense white matter lesions (WML) were scored using the semi-quantitative age related white matter changes (ARWMC) rating scale [32]. Lesions were graded in five regions (frontal, temporal, parieto-occipital, infratentorial, basal ganglia) for each hemisphere ranging from 0 (no lesions) to 3 (confluent affection of entire region). Widening of the Virchow-Robin-spaces (VRS) was scored based on the presence of hypointense foci in the T1-weighted images, according to 0 (no widening of VRS), 1 (few widened foci, restricted to the globus pallidus and putamen), 2 (few widened VRS in basal ganglia and frontal or occipital white matter), 3 (many /marked widened VRS in basal ganglia and frontal or occipital white matter).

### Clinical examination

The patients were thoroughly examined neurologically by an experienced neurologist (CSG) for any clinical symptoms or signs indicating CNS involvement. Additionally, patients were asked for symptoms of daytime sleepiness and the presence of a restless-legs-syndrome (RLS). Patients showing signs of sleep apnoea or needing artificial ventilation support were excluded from the study. The severity of daytime sleepiness was scored by the Epworth sleepiness scale (ESS). The ESS classifies values of 0–10 as “no / moderate sleepiness”, 11–18 as “sleepy” and 19–24 as “very sleepy” [33]. Patients were interviewed for RLS using the Cambridge-Hopkins Questionnaire for ascertainment of RLS (short version 2) [34]. Furthermore the patients were examined for distinct neuropsychological deficits like apraxia, dyscalculia, or dyslexia, signs of Parkinson’s disease, indicators of pyramidal tract lesion as well as for cerebellar and brainstem symptoms and signs. For classification of muscle weakness in DM1 the muscular impairment rating scale (MIRS) was used [35]: MIRS Grade 1 = no muscular impairment, grade 2 = minimal signs, grade 3 = distal weakness but no proximal weakness, 4–5 = proximal weakness. Since a respective scale does not exist for DM2 we used the score in an adapted—yet non-standardised manner- taking into account that muscle weakness in DM2 involves proximal muscles first and that the thumb and deep finger flexors can be involved rather early in the disease [3]. We classified accordingly: Grade 1 = no muscular impairment, grade 2 = minimal signs including myotonia, neck flexor weakness, no proximal weakness, grade 3 = proximal weakness but no distal weakness except for thumb and deep finger flexor weakness, grade 4 = proximal and distal weakness and grade 5 = severe proximal and distal weakness.

#### Neuropsychological testing

All patients were examined by an experienced neuropsychologist (CP) using a standardized neuropsychological test battery. Testing for cognitive impairment included tests for selective attention: d2-Test and Go /no-Go test from the computerized Test for Attentional Performance (TAP) (http://www.psytest.net/index.php?page=Produkte&hl=en_US), and divided attention from TAP, simple reaction ability (tonic and phasic alertness from TAP), non-verbal intellectual ability (from the intelligence test Leistungsprüfungssystem (LPS)-Subtest 3 [36], flexibility of thinking as a form of executive function (Regensburg verbal fluency test (RWT) [37], verbal short-term memory (digit-span forward and backward from Wechsler-Memory Scale (WMS)-R) [38], nonverbal short term memory (blocktapping forward and backward from WMS-R), long-term verbal memory (Rey auditory verbal learning test, Version A(RALVT)), spatial visualisation ability LPS (LPS subtest 7), a general intelligence test (multiple choice vocabulary) and a dementia screening using the clock-drawing test. The sources of normative data for the neuropsychological tests were defined in the statistical manuals of the tests. The neuropsychological findings were classified as pathological or non-pathological according to the normative data only using a threshold determined by 1 standard deviation below the normative mean. In addition, the Beck Depression Inventory-II (BDI-II) was assessed which classifies values of 0 to 11: ormal, 11–19: mild depression, 20–26: median grade depression, >26: marked symptoms of depression.

#### Statistics

All statistical analyses except the SPM exploration were conducted using the SPSS 20 software package (IBM Corporation, NY,USA). Normal distribution of the results was investigated by Kolmogorow-Smirnow testing (p > 0.05).

Associations between MRI, clinical, or neuropsychological results were assessed by Spearman rank correlation analyses. Group differences between the DM groups and healthy controls with respect to MRI results were studied by univariate ANOVAs with subsequent Fisher’s LSD tests for post-hoc correction for multiple comparisons. Mann-Whitney-U tests were used to investigate group differences regarding the ARWMC lesion score, between left / right differences, groups with pathological or non-pathological neuropsychology test results, gender differences or study group.

## Results

### Clinical findings, daytime sleepiness, depression

Comparing the two patient groups (Table 1), DM2 patients had higher depression scores than the DM1 patients (50% of the DM2 patients and 25% of the DM1 patients had Beck’s depression inventory scores > 11). Mild depression was detected in two DM1 and four DM2 patients, and moderate depression in one DM1 and one DM2 patients. Severe depression was found in three DM2 patients. No participant received antidepressants during the study.

Higher grades of daytime sleepiness were reported by the DM1 patients (58% with ESS > 11) compared to the DM2 group (25% exceeding ESS > 11; Table 1).

Muscular impairment as assessed by the MIRS was more severe in DM1 compared to the adapted, non-standardised MIRS in DM2 (Table 1) and was significantly correlated with disease duration in DM1 (ρ = 0.785, P = 0.002).

Restless legs and symptoms indicative for cerebellar involvement were found in DM2 patients only, while cases of unilateral rigidity in the wrist without signs of myotonia were observed in both groups. Clinical and symptomatic details are provided in Table 2.

Age and disease duration were not significantly associated in both groups.

### Neuropsychological testing

Neuropsychological testing revealed major deficits in attention, verbal and non-verbal short term memory, flexibility of thinking and spatial visualization functions in DM1 and DM2 with no significant differences between the groups. The general intelligence level and the long-term verbal memory were not restricted. Table 3 summarizes the results of tests in which at least four DM1 or DM2 patients had pathological results.

**Table 3:** Numbers of abnormal neuropsychological test results in DM1 and DM2.

Test		DM1 (n = 12)	DM2 (n = 16)
**Selective Attention (d2-test)**		5 *(42%)*	4 *(25%)*
**Tonic Alertness**		7 *(58%)*	7 *(44%)*
**Phasic Alertness**		7 *(58%)*	7 *(44%)*
**Go/No-GO reaction time**		6 *(50%)*	6 *(38%)*
**Divided Attention**	reaction time	9 *(75%)*	7 *(44%)*
	missed events	2 *(17%)*	6 *(38%)*
**Flexibility of thinking /Verbal Fluency test / RWT** ^a^	Fluency	4 *(33%)*	5 *(31%)*
	change of categories	8 *(67%)*	8*(50%)*
**Verbal Short Term Memory (digitspan /WMS-R** ^b^ **)**	Numbers forward	3 *(25%)*	4 *(25%)*
	Numbers backward	6 *(60%)*	6 (*38%)*
**Non-verbal Short Term Memory (Block tapping/WMS-R** ^b^ **)**	Forward	7 *(58%)*	12*(75%)*
	Backward	7 *(58%)*	10 *(63%)*
**Auditory Verbal Learning Test (RAVLT** ^c^ **) Long Term Verbal Memory**	course 1	0 *(0%)*	4 *(25%)*
	course 5	1 *(1%)*	0 *(0%)*
	course 6	1 *(1%)*	2 *(12%)*
	delay 7	1 *(1%)*	2 *(12%)*
	repetition 8	1 *(1%)*	1 *(6%)*
**Spatial Visualization Functions(LPS** ^d^ **Subtest 7)**		5 *(42%)*	3 *(19%)*

^a^: RWT Regensburg verbal fluency test;

^b^: WMS Regensburg verbal fluency test;

^c^: RAVLT Rey auditiory verbal learning test,Version A;

^d^: LPS Leistungsprüfsystem.

### MRI findings

#### CNS volumetry

Table 4 shows the volumetry results (group mean values and standard deviation) of the patient groups and healthy controls. In both DM groups significant supratentorial brain atrophy, mainly driven by GM atrophy, was observed. Infratentorially, we detected a tendency for cerebellar atrophy in DM2, which did not reach significance. There was no cervical cord atrophy in DM patients. The lateral ventricles, the 3^rd^. and the 4^rth^ ventricle were significantly widened in both groups. The group mean values of the temporal horn index were higher in both patient groups compared to the healthy controls, yet the differences were not statistically significant.

**Table 4:** Volumetry results within the DM 1 and DM 2 groups and in the healthy controls.

		Controls (n = 33)	DM1 (n = 12)	DM 2 (n = 16)
**Brain WM / ml** ^§^	mean ± SD	593.2 ± 42.8	580.0 ± 1.7	568.9 ± 48.4
	P-value			*^b^P = 0.084*
**Brain GM/ ml** ^§§^	mean ± SD	686.2 ± 35.9	622.9 ± 43.8	630.1 ± 37.8
	P-value		^a^P<0.001**	^b^P<0.001**
**Supratentorial brain volume / ml** ^§§^	mean ± SD	1100.4 ± 46.8	1023.9 ± 62.7	1021.5 ± 50.9
	P-value		^a^P = 0.001**	^b^P<0.001**
**Upper cervical cord area / mm^2^** ^§^	mean ± SD	87.3 ± 7.4	86.1 ± 9.2	90.7± 8.1
**Cerebellum volume / ml** ^§§^	mean ± SD	142.1 ± 9.9	143.8 ± 12.8	135.5 ± 14.3
	P-value		*^c^P = 0.096*	*^b^P = 0.134*
**Brainstem volume / ml** ^§^	mean ± SD	32.7 ± 2.5	31.2 ± 2.8	31.3 ± .3
**Lateral ventricle volume / ml** ^§§^	mean ± SD	16.1 ± 3.9	29.6 ± 14.6	31 ± 9.7
	P-value		^a^P<0.001**	^b^P<0.001**
**3VV / ml** ^§§^	mean ± SD	0.9 ± 0.4	1.8 ± 0.7	1.7 ± 0.8
	P-value		^a^P = 0.001**	^b^P = 0.001**
**4VV / ml** ^§^	mean ± SD	1.5 ± 0.4	2.1 ± 0.6	2.0 ± 0.6
	P-value		^a^P = 0.006**	^b^P = 0.013*
**THI / %**	mean ± SD	1.9 ± 1.0	2.8 ± 2.0	2.6 ± 2.0
	[median; min.-max.]	[2.1; -0.1–3.3]	[2.6; 0.1–6.8]	[1.8; 0.2–6.7]

P = Significance of group differences by univariate ANOVA with post-hoc Fisher’s LSD for multiple comparisons; (*: significant;**: highly significant with P<0.010): a) DM1 compared to healthy controls, b) DM1 compared to healthy controls c) DM1 compared to DM2.

^§^ all volumes and cord area normalized to the intra-cranial cavity volume.

^§§^ corrected for physiological aging.

None of the differences between DM1 and DM2 was statistically significant.

In DM2 patients we found significant positive correlations of the temporal horn index and the 4VV with age (ρ = 0.661, P = 0.007 respectively ρ = 0.621, P = 0.013).

#### Normal distribution and sex-related differences

All MRI results except the total FLAIR lesion load were distributed normally. To provide normality a natural log transformation was applied to the lesion load results. No significant gender related differences were detected.

#### VBM analysis of regional GM and WM atrophy

**Fig 1:**
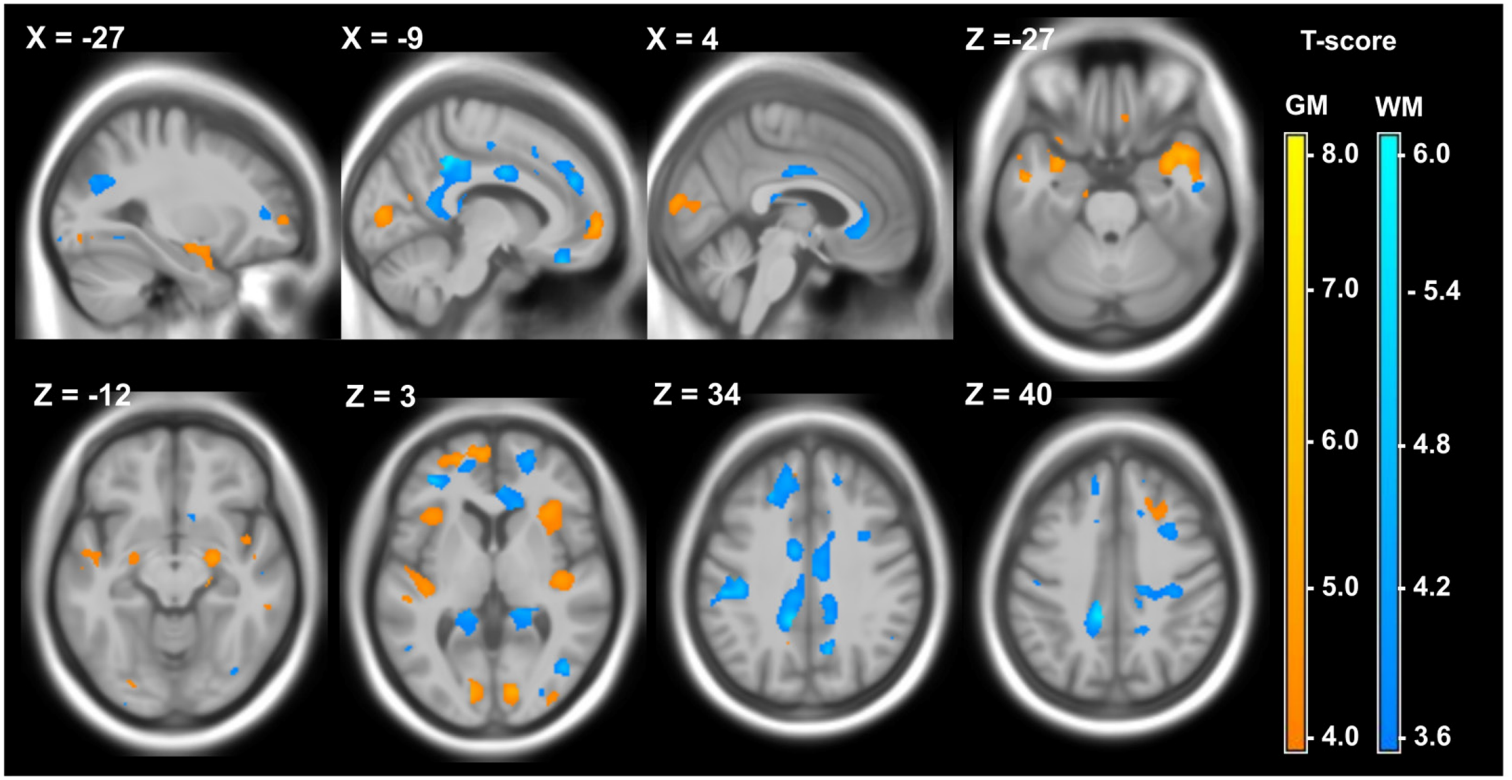
Local atrophy of GM (yellow) and WM (blue) in 12 DM1 patients compared to 28 healthy controls. Voxelwise multiple regression analysis with group and age as covariates. Age was used as a covariate of no interest. Areas with adjusted p at cluster level < 0.05 after FWE correction are shown; X,Y,Z: MNI-coordinates: negative X-values reflect left side and positive X-values right sided location.

**Fig 2:**
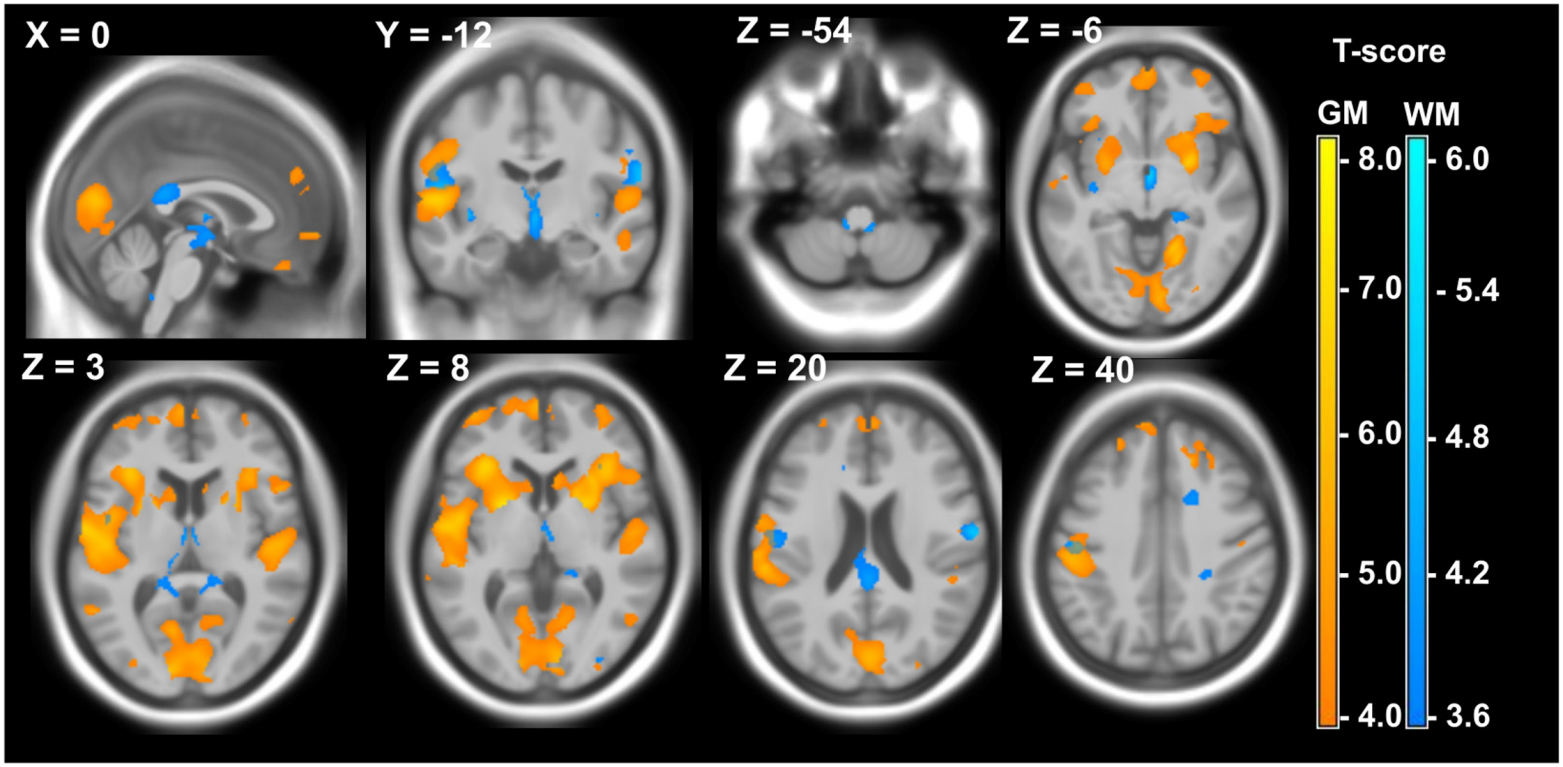
Local atrophy of GM (yellow) and WM (blue) in 15 DM2 patients compared to 28 healthy controls. Voxelwise multiple regression analysis with group and age as covariates. Age was used as a covariate of no interest. Areas with adjusted p at cluster level < 0.05 after FWE correction are shown; X,Y,Z: MNI-coordinates: negative X-values reflect left side and positive X-values right sided location.

VBM comparing patients with controls showed significant atrophy of GM and WM in DM1 and DM2, with partly different local distribution of atrophy. Details of the VBM results are presented in Figs 1 and 2. Numerical cluster details and MNI coordinates are presented S1 Table and S2 Table.

In the DM1 group significant areas of GM atrophy were found in both hemispheres in the lingual gyrus and cuneus, insula, the middle und superior temporal gyrus extending to the precentral gyrus, and the medial frontal gyrus (Fig 1). In this group also the sub-cortical GM was involved by significant bilateral atrophy of the putamen and caudate head. White matter atrophy was observed in DM1 in posterior corpus callosum, bilaterally in the mediodorsal nucleus and pulvinar of the thalamus and left-hemispheric WM adjacent to the pre- and post-central gyrus. Thus, in DM1, structures belonging to the motor system (motor cortex, thalamus/putamen) were affected by atrophy.

The DM2 patients showed similar patterns of bilateral GM atrophy in the cuneus and temporal / insular regions in comparison to DM1 (Fig 2). In contrast to DM1, we found bilateral GM atrophy in the superior temporal cortex and the anterior temporal poles. Medial frontal lobe atrophy was mainly found in the left hemisphere. Furthermore, in the DM2 group, the bilateral amygdala was affected.

Bilateral WM atrophy, was detected in DM2 predominantly in the cingulate, and furthermore in the subgyral WM of the medial frontal cortex and the primary somatosensory cortex (Brodmann areas BA2, BA40).

Overall, we found larger clusters of GM atrophy in DM1 compared to DM2. In contrast, clusters of WM atrophy were larger in DM2 compared to DM1 (Figs 1 and 2, S1 Table and S2 Table). Although not statistically significant, these finding were in accordance, with the results of the global volumetry, which showed lower GM volumes in DM1 and lower WM volumes in DM2 (Table 4).

#### Correlations of MRI findings and MIRS

Although marked brain atrophy and ventricular expansion were detected in both DM subgroups, we found relatively few significant correlations between these parameters and distinct neurological or cognitive deficits: In the DM1 group the total GM volume was significantly inversely correlated with 3VV, 4VV and the lateral ventricular volume (Spearman correlation: ρ = -0.580, P = 0.048; ρ = -0.601, P = 0.039; ρ = -0.839, P = 0.001) indicating a significant proportion of subcortical GM atrophy, which corresponded to the putamen and caudate atrophy in DM1 detected by VBM (see above, and Fig 1). The MIRS in DM1 was inversely correlated with the supratentorial brain volume (ρ = -0.728, P = 0.007).

#### Associations between depression and brainstem atrophy

**Fig 3:**
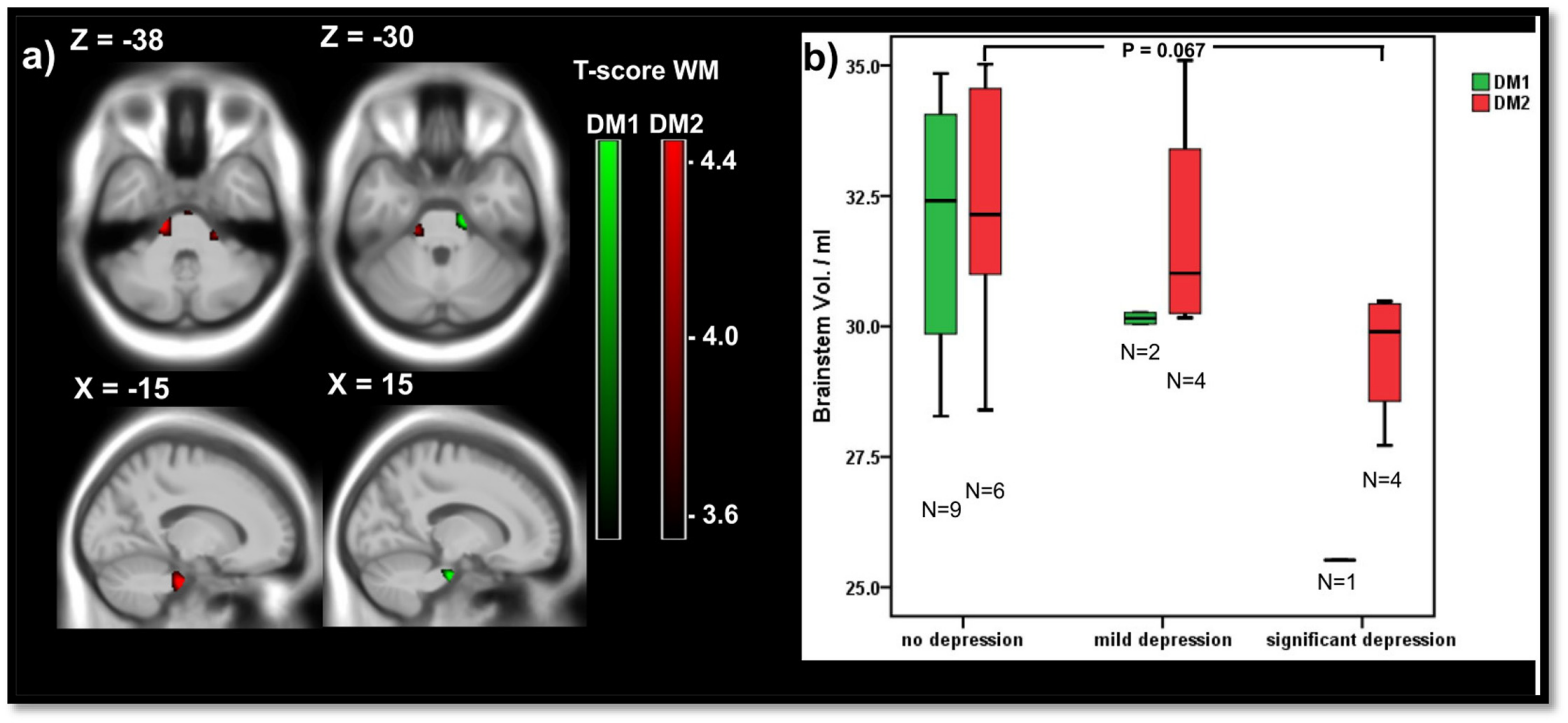
Depression and brainstem volume. a) Negative correlations between white matter and the depression grade in 12 DM1 (green) and 15 DM2 (red) patients: voxelwise multiple regression with the depression score (BDI-II) as covariate; areas with uncorrected p<0.001 at peak level are shown; X,Y,Z: MNI-coordinates: negative X-values reflect left side and positive X-values right sided location. b) Brainstem volume of DM1 and DM2 patients in dependency of the depression grade: no depression: score < = 11; mild depression: score 12–19; significant depression: score > 20; significance of group differences by Mann-Whitney-U tests.

Signs of depression were observed in both patient groups and were more pronounced in DM2 (Table 1 *)*. Group comparisons between patients with different depression grades (no depression, mild depression, severe depression) revealed a trend in DM2 for brainstem atrophy in severe depression compared to patients without depression (Mann-Whitney-U testing p = 0.067; see Fig 3b). The DM1 results were in accordance with this trend, but the small numbers of patients with clinically relevant depression prevented a statistical analysis in this group. In a pooled data analysis of DM1 and DM2 the difference between the brainstem volume of patients with significant depression compared to no depression was significant (P = 0.030, data not shown).

Voxelwise regression analysis between WM and the depression score was done to further elucidate the localization of brainstem areas in which WM volume was associated with depression. The analyses revealed consistent WM clusters localized bilaterally in the pons at the level of the cerebellar peduncles in both groups and were more pronounced in DM2 (significant at uncorrected peak level with P<0.001; Fig 3a). Because consistent WM patterns were involved in both groups, we present summarized cluster details of the pooled DM1 and DM2 data in S3 Table.

#### Associations between daytime sleepiness in DM and brain atrophy

**Fig 4:**
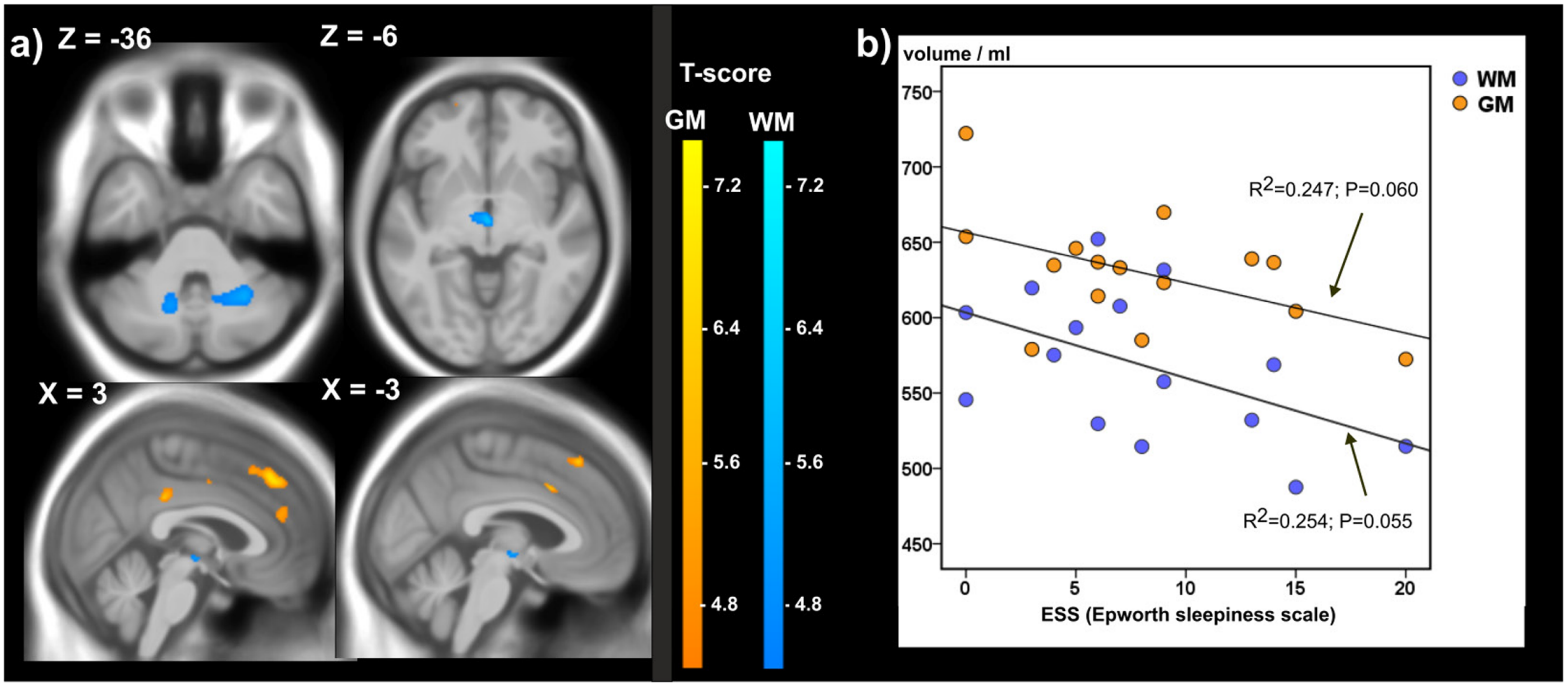
ESS and brain grey and white matter in DM2. a) Significant negative correlations between GM and WM and Epworth sleepiness score in 15 DM2 patients detected by voxelwise multiple regression analysis. Age was used as a covariate of no interest. Clusters with FWE corrected cluster p-values < 0.07 are depicted; MNI coordinates: negative X-values reflect left side and positive X-values right sided location. b) Scatterplot of global brain GM and WM in dependency on ESS; the lines and annotations reflect the results of the linear regression analysis.

DM patients in both disease groups suffered from distinctive daytime sleepiness (Table 1). Although DM1 patients were more affected, correlation analyses between the global CNS volumes and the ESS in both groups revealed weak negative associations between global GM and WM and the ESS only in the DM2 group (Spearman correlation: GM ρ = -0.392, P = 0.148; WM ρ = -0.460, P = 0.084, and Fig 4b).

The voxelwise regression analysis of grey and white matter in DM1 and DM2 with the ESS score and age as covariates resulted in significant negative correlations between GM and ESS in the mediofrontal cortex (Brodman area 8) and between WM and ESS in the middle cerebellar peduncles bilaterally and in the superior pons/midbrain in the DM2 group (Fig 4a, S4 Table). In Fig 4b the associations in DM2 between ESS and global brain WM, respectively GM, are displayed for clarification. Although in DM1 a higher percentage of patients was shown to be affected by daytime sleepiness, we detected no significant correlations between ESS and GM or WM in this group.

#### Associations of neuropsychological results with regional CNS volumes

**Fig 5:**
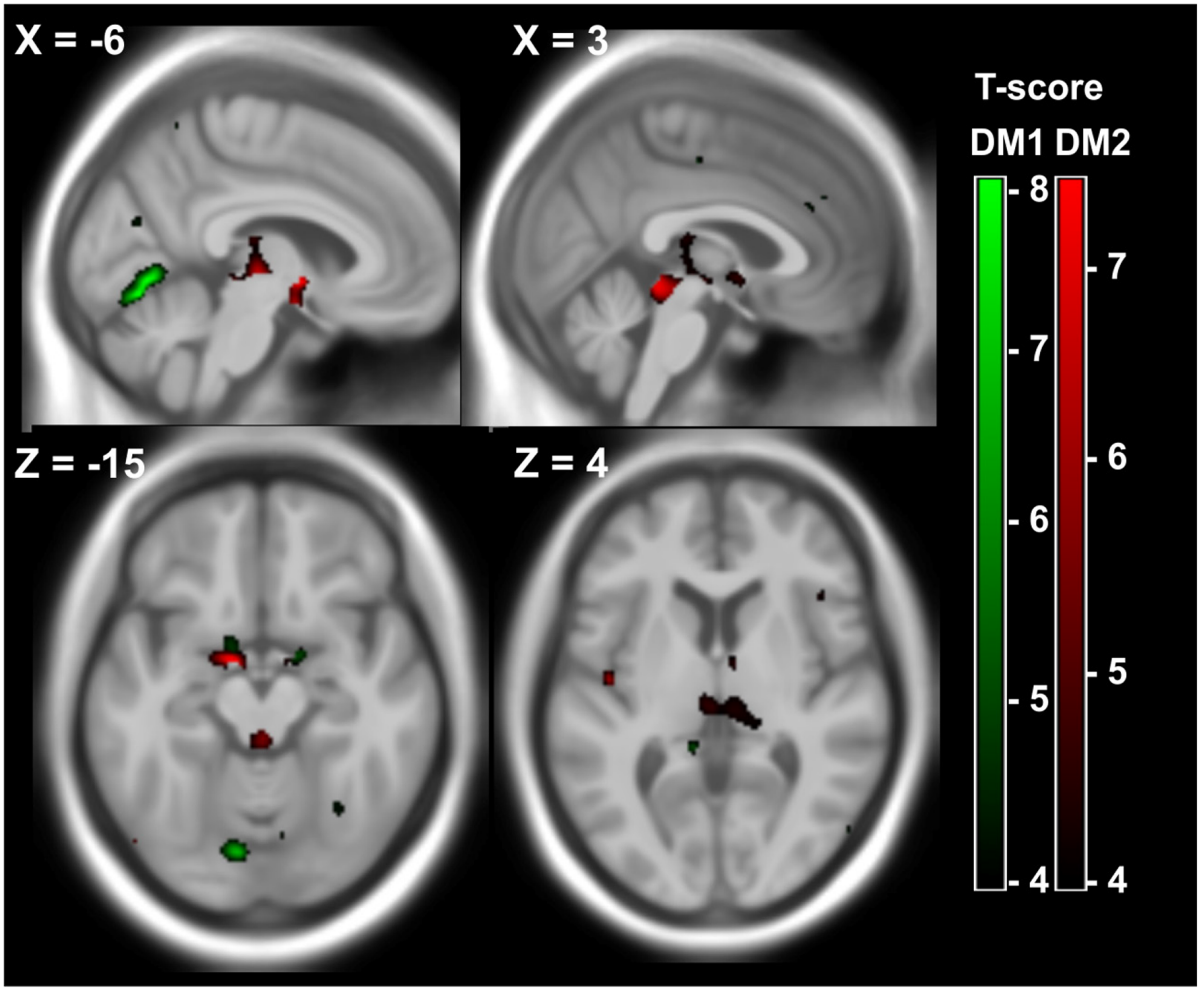
Flexibility of thinking and brain GM. Areas of significant negative correlations between GM volume and flexibility of thinking (RWT semantic verbal fluency with change of categories) in 12 DM1 patients (green) and 15 DM2 patients (red). Voxelwise multiple regression analysis with the test outcome (pathologic / normal) and age as covariates. Age was used as a covariate of no interest. Areas with adjusted p at cluster level < 0.05 after FWE correction are shown; X,Y,Z: MNI-coordinates: negative X-values reflect left side and positive X-values right sided location.

We examined voxelwise regression analyses for GM and WM using the results of the most relevant neuropsychological tests as covariates (Table 3). These were: Attention (reflected by tonic and phasic alertness), flexibility of thinking (RWT test with change of categories), non-verbal short-term memory (WMS-R: block-tapping-forward and-backwards), and verbal short term memory (WMS-R, repetition of numbers backward). The only statistically significant association was an inverse correlation between GM volume and flexibility of thinking, when using the group classification according to pathological or normal neuropsychological test outcome and age as covariates. In DM1, significant clusters were detected in the left hemispheric secondary visual cortex (medio-parietal cortex: BA18, BA19). In DM2 significant clusters were located in the periaqueductal grey matter, in parts of the midbrain, the bilateral thalamus (pulvinar), in the bilateral parahippocampal gyrus (BA34) and in the anterior cingulate (BA25), (Fig 5 and S5 Table). All other associations between voxelwise brain volumes and neuropsychological parameters were non-significant.

#### Brain white matter lesions in DM1 and DM2

Considerable numbers of hyperintense WM lesions in DM1 and DM2 patients were found only in patients older than 40 years, without signs of asymmetric lesions distribution or gender specific differences in any subgroup (S6 Table).

In DM1 patients, WM lesions were mainly seen as focal lesions in frontal, parietal or occipital regions. Young DM1 patients showed widened Virchow-Robin-spaces especially in the parieto-occipital and frontal WM regions, but very few WM lesions. Within the DM2 group, we found heterogeneous lesion patterns with 11 patients having only a minor lesion load (< = 1 ml) and 5 patients showing large confluent WM lesions (8 to 100 ml) with a leukodystrophy-like pattern focussed in frontal and parieto-occipital WM. Widened VRS were not observed in the DM2 group. The presence of large leukodystrophy-like WM lesions in the DM2 patient group was associated with a higher prevalence for neurological CNS symptoms (cerebellar symptoms, rigor of the wrist, dysmetria, dysdochokinesia, restless legs syndrome), with four of five affected patients (80%) showing neurological CNS symptoms, while of the remaining 11 DM2 patients without confluent WM lesions only three patients (27%) had CNS symptoms. This observation did not reach statistical significance in the chi-square test (p = 0.067).

We detected no other significant relations of the WM lesion scores with any of the other parameters.

## Discussion

We present a detailed analysis of morphological brain abnormalities in both types DM1 and DM2 as shown by MRI at 3 Tesla, focussing on volumetry and VBM abnormalities. Our study revealed several new aspects. First, in contrast to previous studies (8, 11) we found a different relation of GM and WM loss with more pronounced cortical and subcortical GM loss than WM loss in both DM types, Second, our study showed that EDS (in the absence of obstructive sleep apnoea) and depression are major CNS symptoms not only in DM1, but also in DM2. Third, we could demonstrate that EDS and depression are likely to be related to distinct structural findings as indicated by abnormalities of sleep regulation structures and a trend for a correlation of the severity of depression with brainstem atrophy in DM2. Fourth, volumetry of the upper spinal cord as performed for the first time in our study revealed no abnormalities in DM1 and DM2. We confirmed WM affection in both DM types with a higher degree of abnormal WM findings (atrophy and hyper-intense WM lesions) in DM2. Neuropsychologically, deficits in flexibility of thinking, attention, short-term memory and visuo-spatial abilities were found in both groups, which was confirmatory to several studies [5, 8, 14–16]. Hyperintense WM lesions occurred in both DM groups preferably at ages higher than 40 years, but had only a limited clinical significance in our study.

### Local GM and WM atrophy in DM1 and DM2

Structural MRI showed considerable affection of brain grey matter and white matter in both DM1 and DM2 by using different independent methods. Substantial supratentorial global atrophy in both DM groups was mainly driven by GM atrophy and to a lesser extent by WM atrophy, where GM decrease was more pronounced in DM1 contrasting to more WM atrophy in DM2 (Table 4). For the first time, we investigated spinal atrophy in DM and found no atrophy of the upper cervical cord but mild involvement of the cerebellum in DM2.

The VBM analysis revealed substantial cortical GM affection in both patient groups in the lingual gyrus, cuneus, insula, middle and superior temporal and frontal lobes where as in a previous studiy mainly frontal and parietal regions and in particular the pre- and postcentral gyrus and the supplementary motor cortex were described to be affected, while temporal lobes were spared [8]. We detected specific cortical differences between DM1 and DM2, namely GM atrophy of the motor-cortex in DM1 and GM loss in the anterior poles of the temporal lobes in DM2.

Subcortically, in DM1 mainly the putamen-as described in [8], and caudate head showed GM atrophy, but also WM atrophy of the thalamus and splenium of the corpus callosum could be shown. As a new finding, GM atrophy of the amygdala was shown in DM2. In DM2 the cingulate and the subgyral WM of the medial frontal cortex and the primary somatosensory cortex (Brodmann areas BA2, BA40) showed WM loss.

A previous study revealed more widespread and more pronounced WM than GM loss in DM1 and DM2 affecting WM of every lobe, the fornices, the whole corpus callosum, and the brainstem at the level of the pons as well as WM along the middle cerebellar peduncles and cerebellar WM [8]. The pattern of WM loss was similar in DM1 and DM2 in that study.

Caso et al. demonstrated cortical GM atrophy of the pre- and postcentral, supplementary motor orbitofrontal, medial and dorsolateral frontal, insular, cingulated, hippocampal, parietal, and occipital cortex and of putamen, thalamus and caudate nucleus bilaterally in a group of adult DM1 patients resembling the pattern of GM loss in our DM1 patients in many aspects. WM pathology consisted of widespread WM hyperintensities and involvement of all major WM tracts using DTI, but voxelwise WM atrophy by VBM was not assessed [11]. The authors interpreted the distribution and extent of WM changes as suggestive of GM loss evolving secondary to WM pathology.

For different study designs we cannot compare these results directly to our VBM data in DM1 patients. Discrepancies of our results compared to other groups might be explained by different methodical approaches, i.e. lesion filling prior to quantitative analysis, but also by clinical heterogeneity of different study cohorts. However, the amount and distribution of GM loss as compared to WM loss in our study could be interpreted in favour of at least partially primary disease related GM pathology in addition to possible age related changes in DM1 and DM2. Predominant age related GM atrophy was suggested recently on the basis of comparative MRI data in adult and juvenile or congenital DM1 patients [6, 11] and by comparing DM1 patients and healthy controls (14). Further studies will be needed to clarify the interrelations of WM and GM pathology and whether these relations remain stable during the disease or change with age and duration of the disease.

### Structural-functional correlations

#### Central motor impairment in DM1

Significant correlations of the MIRS with global brain atrophy and evidence for regional atrophy along the central motor structures at different levels (motor / supplemental motor cortex, subgyral WM, basal ganglia) pointed to structural central correlates of reduced/impaired planning and execution of motor functions/actions in DM1 as postulated previously [39, 40]. Consistently, a previous study revealed reduced fractional anisotropy of the corticospinal tract at the level of the internal capsule and pons correlating with impaired motor performance only in DM1, thus giving further evidence of central motor dysfunction in adult DM1 [8] whereas in childhood and adolescent DM1 the myelin of the corticospinal tracts was shown to be relatively preserved in DTI studies [10].

#### Neuropsychology and cognition

We detected relevant correlations between local brain volume and neurocognitive findings in DM1 and DM2 merely for executive functions, namely flexibility of thinking / phonemic fluency. VBM showed predominant central atrophy in DM2 (periaqueductal GM, midbrain,and amygdala) and atrophy of the left secondary visual cortex in DM1 in association with impaired flexibility of thinking (Fig 5 and S5 Table). We speculate, that degraded flexibility of thinking in DM2 may be related to the affection of deep GM in particular of associative interconnections passing through and being regulated by deep grey matter nuclei [41]. Furthermore, the cortical area involved in DM1 is functionally related to complex language processing and visual functions [42].

We found no relation of cognitive impairment to frontal lobe affection as suggested by previous PET and MRS studies [15, 17] and a recent neuropsychological study [43]. Differences in sensitivity of the neuroimaging techniques employed might be one of the possible explanations for this discrepancy. On the other hand, a recent study using resting-state functional MRI in DM1, revealed that neuropsychologic deficiencies in the DMs may result from modification of scattered neuronal networks rather than local tissue affection. Notably, in this study, an association between the connectivity in the basal ganglia and the severity of psychotic symptoms in DM1 has been discovered [19].

Thus, our results suggest a role of subcortical deep GM in DM2 and the visual cortex in DM1 in the reduction of executive functions. In contrast, a different recent DM1 study revealed a correlation of cognitive impairment, in particular impaired phonemic fluency, reasoning and problem solving, and memory functions with microstructural WM abnormalities (interhemisperic, limbic, corticospinal and associative WM pathways) but no relation to GM loss [11]. Thus, there are still inconsistent structural-functional correlations in the myotonic dystrophies that need further clarification.

#### Depression and brainstem atrophy

Depression in DM1 and DM2 has been suspected to be either reactive or a result of structural or functional changes in the brain (8). The vulnerability of the brainstem in DM1 has previously been described in a VBM study and histopathologically, through detection of oxidative products and neurofibrillary tangles in the brainstem [8, 20]. In the present MRI study we detected associations between WM brainstem atrophy at the level of the basal pons and the middle cerebellar peduncles in both groups and the depression score in DM1 and DM2, which reached statistical significance only in DM2 (Fig 3a and 3B). Thus, our results corroborate the hypothesis, that depression in DM1 and DM2 has at least in part a pathomorphological correlate, which involves the pons and probably the middle cerebellar peduncles and is not only a reactive phenomenon. The pontine nuclei and projection fibers are functionally highly eloquent tissues. There are indications that the communication between cerebral and cerebellar cortical activity connects the pontine nuclei with the limbic system, which can thus be involved in depression and emotional control [44]. On the other hand, mesopontine nuclei of the brainstem, like the raphe nuclei, are part of the serotonergic system, which is essential for depression and sleep regulation [45].

#### Daytime sleepiness and regional brain atrophy

Daytime sleepiness, which is one of the major non-motor symptoms affecting the patients’ quality of life, has been interpreted as secondary to progression of respiratory muscle weakness and with increasing evidence as a result of CNS dysfunction [8, 46]. Sleepiness in DM1 was inversely associated with WM fiber integrity in the superior longitudinal fascicle and cingulum in a study using DTI [9]. We demonstrated significant negative correlations between ESS and GM in the DM2 group, in the mediofrontal cortex (Brodman area 8) and between WM and ESS in the middle cerebellar peduncles bilaterally and in the superior pons/midbrain in the DM2 group. The lack of a comparable structural-functional association in the DM1 patients, who were more afflicted by excessive daytime sleepiness, is still unclear. Yet, the brain structures involved in the DM2 group, especially the right medio-frontal cortex and the midbrain have been shown by neuroimaging studies to be partly involved in the complex system of sleep and sleep regulation [47, 48] thus supporting the hypothesis that structural CNS alterations may be involved in daytime sleepiness in the DMs.

## Conclusion

In conclusion our results revealed widespread cortical and subcortical GM and WM atrophy in DMs specifically with affection of the central motor pathways in DM1 and limbic structures in DM2. We demonstrated morphological correlates for depression, daytime sleepiness and reduced executive functions in the DMs. The complexity of our results supports the hypothesis that CNS symptoms in DM are related to impairment of complex neuronal networks rather than to focal neurodegeneration. However, the sample sizes are relatively small which limits the interpretation of our results. Confirming previous studies, our investigations showed that only a small part of the cognitive symptoms in DM1 and DM2 could be represented by the morphometric techniques. The discrepancies between the different studies including ours may be attributed to differences in study design and population (age, duration of the disease, number of patients included), and clinical and morphological variety and heterogeneity of the disease due to mosaicism and instability of the mutation and individual genetic and epigenetic factors. As previously proposed further clinical, molecular, neuropatholoical and imaging studies are necessary to understand the complex nature of CNS affection in DMs. Longitudinal MRI studies are warranted to elucidate the natural course of CNS involvement in DM1 and DM2, and to further differentiate neurodevelopmental and neurodegenerative processes [49].

## Supporting Information

S1 Table GM and WM atrophy in DM1 relative to healthy controls. Areas of significant atrophy of brain GM and WM in DM1 compared to healthy controls by multiple regression analysis with type and age as covariates; areas with adjusted p at cluster level < 0.05 after FWE correction with local maxima more than 8 mm apart are shown; Central brain details reflect small volume correction in a central sphere of radius 30 mm: negative X-values reflect left side and positive X-values right sided location.
(DOCX)

S2 Table GM and WM atrophy in DM2 relative to healthy controls. Areas of significant atrophy of brain GM and WM in DM2 compared to healthy controls by multiple regression analysis with type and age as covariates; areas with adjusted p at cluster level < 0.05 after FWE correction with local maxima more than 8 mm apart are shown; Central brain details reflect small volume correction in a central sphere of radius 30 mm: negative X-values reflect left side and positive X-values right sided location.
(DOCX)

S3 Table Depression and brain WM. Areas of significant correlations between brain WM and depression score in DM (pooled data of DM1 and DM2) by voxelwise multiple regression analysis; areas with adjusted p at cluster level < 0.07 after FWE correction with local maxima more than 4 mm apart are shown; MNI coordinates: negative X-values reflect left side and positive X-values right sided location.
(DOCX)

S4 Table ESS and brain GM and WM in DM2. Areas of significant correlations between brain GM and WM and Epworth sleepiness score in DM2 by voxelwise multiple regression analysis with age as covariate; areas with adjusted p at cluster level < 0.07 after FWE correction with local maxima more than 8 mm apart are shown; MNI coordinates: negative X-values reflect left side and positive X-values right sided location.
(DOCX)

S5 Table Flexibility of thinking and brain GM. Areas of significant correlations between brain GM and flexibility of thinking (RWT test with change of categories) in DM1 and DM2 by voxelwise multiple regression analysis with type and age as covariates; areas with adjusted p at cluster level < 0.05 after FWE correction with local maxima more than 4 mm apart are shown; negative X-values reflect left side and positive X-values right sided location.
(DOCX)

S6 Table Lesion grading in DM1, DM2 and the healthy control group. ARWMC (age related white matter changes) score, the VRS (Virchow-Robin-spaces) score and the total FLAIR lesion load in DM1, DM2 and the healthy control group. Significance of group differences by Mann-Whitney-U tests (P > 0.050: not significant [n.s.], P < 0.050: significant, P <0.010: highly significant [bold]).
(DOCX)
