# Supplementary material for: Cortical and Subcortical Grey and White Matter Atrophy in Myotonic Dystrophies Type 1 and 2 Is Associated with Cognitive Impairment, Depression and Daytime Sleepiness
Source: PLoS One. 2015 Jun 26;10(6):e0130352. doi: 10.1371/journal.pone.0130352 (PMC4482602; doi:10.1371/journal.pone.0130352)
Supplement: S1 Table — Areas of significant atrophy of brain GM and WM in DM1 compared to healthy controls by multiple regression analysis with type and age as covariates; areas with adjusted p at cluster level < 0.05 after FWE correction with local maxima more than 8 mm apart are shown; Central brain details reflect small volume correction in a central sphere of radius 30 mm: negative X-values reflect left side and positive X-values right sided location. (DOCX) [file pone.0130352.s001.docx]

**S1 Table: GM and WM atrophy in DM1 relative to healthy controls.**

Areas of significant atrophy of brain GM and WM in DM1 compared to healthy controls by multiple regression analysis with type and age as covariates; areas with adjusted p at cluster level < 0.05 after FWE correction with local maxima more than 8 mm apart are shown; Central brain details reflect small volume correction in a central sphere of radius 30 mm: negative X-values reflect left side and positive X-values right sided location.

| **GREY MATTER** | | | | | | | | |
| --- | --- | --- | --- | --- | --- | --- | --- | --- |
| Region | MNI coordinates | | | cluster level | | peak level | | |
|  | X | Y | Z | p(FWE-corr.) | equiv. cluster size (voxels) | T-score | equiv. Z-score | p(uncorr.) |
| Lentiform Nucleus / Putamen | -22.5 | 3 | 4.5 | 8.9E-16 | 15980 | 7.28 | 5.70 | 6.0E-09 |
| Superior Temporal Gyrus / BA 22 | -57 | -9 | 6 |  |  | 6.64 | 5.35 | 4.3E-08 |
| Insula / BA 13 | -33 | 24 | 4.5 |  |  | 6.64 | 5.35 | 4.4E-08 |
| Middle Temporal Gyrus / BA 20 | 55.5 | -39 | -15 | 4.6E-01 | 245 | 6.94 | 5.52 | 1.7E-08 |
| Lingual Gyrus / BA 19 | 21 | -60 | -1.5 | 6.8E-12 | 10486 | 6.74 | 5.41 | 3.2E-08 |
| Lingual Gyrus / BA 17 | 9 | -88.5 | -3 |  |  | 6.30 | 5.16 | 1.2E-07 |
| Cuneus / BA 18 | -7.5 | -85.5 | 12 |  |  | 6.18 | 5.09 | 1.8E-07 |
| Lentiform Nucleus / Putamen | 27 | 1.5 | -1.5 | 1.5E-07 | 5329 | 6.69 | 5.38 | 3.7E-08 |
| Lentiform Nucleus / Putamen | 19.5 | 7.5 | 4.5 |  |  | 6.15 | 5.07 | 2.0E-07 |
| Claustrum / * | 28.5 | 15 | 4.5 |  |  | 6.13 | 5.06 | 2.1E-07 |
| Transverse Temporal Gyrus / BA 41 | 49.5 | -21 | 10.5 | 6.2E-06 | 3714 | 5.93 | 4.94 | 3.9E-07 |
| Transverse Temporal Gyrus / BA 41 | 48 | -30 | 12 |  |  | 5.82 | 4.87 | 5.5E-07 |
| Precentral Gyrus / BA 6 | 48 | -16.5 | 36 |  |  | 4.40 | 3.92 | 4.4E-05 |
| Middle Frontal Gyrus / BA 10 | 34.5 | 55.5 | -1.5 | 1.6E-11 | 10003 | 5.72 | 4.81 | 7.6E-07 |
| Medial Frontal Gyrus / BA 10 | -3 | 60 | 4.5 |  |  | 5.69 | 4.79 | 8.2E-07 |
| Middle Frontal Gyrus / BA 10 | -30 | 55.5 | 13.5 |  |  | 5.51 | 4.68 | 1.5E-06 |
| Middle Temporal Gyrus / BA 21 | 49.5 | -6 | -16.5 | 6.0E-03 | 1312 | 5.70 | 4.80 | 8.0E-07 |
| Middle Temporal Gyrus / BA 21 | 48 | 9 | -27 |  |  | 4.70 | 4.13 | 1.8E-05 |
| Middle Temporal Gyrus / BA 21 | 52.5 | -16.5 | -19.5 |  |  | 4.36 | 3.89 | 4.9E-05 |
| Superior Temporal Gyrus / BA 22 | 57 | -61.5 | 12 | 2.5E-02 | 920 | 4.56 | 4.04 | 2.7E-05 |
| Middle Temporal Gyrus / BA 37 | 52.5 | -64.5 | 1.5 |  |  | 4.34 | 3.88 | 5.3E-05 |
| Inferior Temporal Gyrus / * | 42 | -66 | -4.5 |  |  | 4.08 | 3.68 | 1.2E-04 |
| **GM; central brain details (local maxima more than 4mm apart)** | | | | | | | | |
| Caudate / Caudate Body | -22.5 | 3 | 4.5 | 5.2E-05 | 2019 | 7.28 | 5.70 | 6.0E-09 |
| Lentiform Nucleus / Putamen | -7.5 | 12 | 7.5 |  |  | 4.62 | 4.08 | 2.3E-05 |
| Lentiform Nucleus / Putamen | -25.5 | 12 | -9 |  |  | 4.55 | 4.03 | 2.8E-05 |
| Lentiform Nucleus / Putamen | 27 | 1.5 | -1.5 | 1.6E-04 | 1666 | 6.69 | 5.38 | 3.7E-08 |
| Lentiform Nucleus / Putamen | 19.5 | 7.5 | 4.5 |  |  | 6.15 | 5.07 | 2.0E-07 |
| Lentiform Nucleus / Putamen | 27 | 10.5 | 6 |  |  | 5.82 | 4.87 | 5.5E-07 |
| Caudate / Caudate Head | 25.5 | 15 | 4.5 |  |  | 5.62 | 4.75 | 1.0E-06 |
| Lentiform Nucleus / Putamen | 7.5 | 16.5 | 3 |  |  | 4.92 | 4.29 | 9.0E-06 |
|  | 24 | 15 | -9 |  |  | 4.89 | 4.27 | 9.8E-06 |
|  |  |  |  |  |  |  |  |  |
| **WHITE MATTER** | | | | | | | | |
| Extra-Nuclear | 3 | -6 | -6 | 4.7E-04 | 2535 | 5.06 | 4.38 | 5.9E-06 |
| Thalamus | 3 | -16.5 | 0 |  |  | 4.37 | 3.90 | 4.8E-05 |
|  | 0 | -12 | 6 |  |  | 4.20 | 3.77 | 8.0E-05 |
| Postcentral Gyrus | -51 | -12 | 12 | 1.9E-02 | 1120 | 4.77 | 4.18 | 1.4E-05 |
| Precentral Gyrus | -48 | -18 | 40.5 |  |  | 4.65 | 4.10 | 2.1E-05 |
| Precentral Gyrus, adjecent to BA13 | -43.5 | -3 | 9 |  |  | 3.97 | 3.60 | 1.6E-04 |
| **WM; central brain details (local maxima more than 4mm apart)** | | | | | | | | |
| Extra-Nuclear WM | 3 | -6 | -6 | 1.2E-04 | 2530 | 5.06 | 4.38 | 5.9E-06 |
| Midbrain | 3 | -16.5 | 0 |  |  | 4.37 | 3.90 | 4.8E-05 |
| Thalamus | 0 | -12 | 6 |  |  | 4.20 | 3.77 | 8.0E-05 |
| Parahippocampal Gyrus | 18 | -34.5 | -3 |  |  | 4.16 | 3.75 | 9.0E-05 |
| Corpus Callosum | 1.5 | -37.5 | 19.5 |  |  | 4.14 | 3.73 | 9.7E-05 |
| Thalamus/ Medial Dorsal Nucleus | -3 | -12 | 10.5 |  |  | 4.09 | 3.69 | 1.1E-04 |
| Thalamus/ Medial Dorsal Nucleus | 4.5 | -15 | 9 |  |  | 3.97 | 3.60 | 1.6E-04 |
| Thalamus/ Medial Dorsal Nucleus | -7.5 | -24 | 9 |  |  | 3.94 | 3.58 | 1.7E-04 |
| Corpus Callosum | -13.5 | -37.5 | 10.5 |  |  | 3.91 | 3.55 | 1.9E-04 |
| Corpus Callosum | -6 | -27 | 18 |  |  | 3.86 | 3.52 | 2.2E-04 |
| Thalamus/ Medial Dorsal Nucleus | 4.5 | -10.5 | 10.5 |  |  | 3.82 | 3.49 | 2.5E-04 |
| Thalamus/ Medial Dorsal Nucleus | -4.5 | -16.5 | 10.5 |  |  | 3.82 | 3.49 | 2.5E-04 |
| Thalamus / Pulvinar | -10.5 | -28.5 | 9 |  |  | 3.79 | 3.46 | 2.7E-04 |
| Thalamus / Pulvinar | 15 | -36 | 10.5 |  |  | 3.79 | 3.46 | 2.7E-04 |
| Extra-Nuclear WM | -3 | -22.5 | 0 |  |  | 3.71 | 3.40 | 3.4E-04 |
| Corpus Callosum | -4.5 | -22.5 | 19.5 |  |  | 3.67 | 3.37 | 3.8E-04 |
