# Supplementary material for: Cortical and Subcortical Grey and White Matter Atrophy in Myotonic Dystrophies Type 1 and 2 Is Associated with Cognitive Impairment, Depression and Daytime Sleepiness
Source: PLoS One. 2015 Jun 26;10(6):e0130352. doi: 10.1371/journal.pone.0130352 (PMC4482602; doi:10.1371/journal.pone.0130352)
Supplement: S2 Table — Areas of significant atrophy of brain GM and WM in DM2 compared to healthy controls by multiple regression analysis with type and age as covariates; areas with adjusted p at cluster level < 0.05 after FWE correction with local maxima more than 8 mm apart are shown; Central brain details reflect small volume correction in a central sphere of radius 30 mm: negative X-values reflect left side and positive X-values right sided location. (DOCX) [file pone.0130352.s002.docx]

**S2 Table: GM and WM atrophy in DM2 relative to healthy controls.**

Areas of significant atrophy of brain GM and WM in DM2 compared to healthy controls by multiple regression analysis with type and age as covariates; areas with adjusted p at cluster level < 0.05 after FWE correction with local maxima more than 8 mm apart are shown; Central brain details reflect small volume correction in a central sphere of radius 30 mm: negative X-values reflect left side and positive X-values right sided location.

| **GREY MATTER** | | | | | | | | |
| --- | --- | --- | --- | --- | --- | --- | --- | --- |
| Region | MNI coordinates | | | cluster level | | peak level | | |
|  | X | Y | Z | p(FWE-corr.) | equiv. cluster size (voxels) | T-score | equiv. Z-score | p(uncorr.) |
| Insula / BA 13 | -36 | -24 | 12 | 1.7E-08 | 3183 | 6.22 | 5.17 | 1.2E-07 |
| Middle Temporal Gyrus / BA 21 | -52.5 | -1.5 | -18 |  |  | 6.18 | 5.14 | 1.3E-07 |
| Postcentral Gyrus / BA 40 | -57 | -31.5 | 18 |  |  | 5.88 | 4.96 | 3.5E-07 |
| Anterior Cingulate / BA 10 | -4.5 | 57 | 0 | 3.3E-04 | 973 | 6.16 | 5.14 | 1.4E-07 |
| Superior Frontal Gyrus / BA 10 | -12 | 57 | -3 |  |  | 5.39 | 4.64 | 1.7E-06 |
| Middle Frontal Gyrus / BA 10 | -28.5 | 52.5 | 1.5 |  |  | 4.63 | 4.12 | 1.9E-05 |
| Middle Temporal Gyrus / BA 39 | 36 | -75 | 15 | 4.3E-02 | 252 | 5.94 | 5.00 | 2.8E-07 |
| Cuneus / BA 17 | 13.5 | -84 | 0 | 7.3E-04 | 837 | 5.89 | 4.97 | 3.4E-07 |
| Cuneus / BA 17 | 13.5 | -85.5 | 10.5 |  |  | 5.00 | 4.38 | 5.9E-06 |
| Cuneus / BA 18 | 4.5 | -91.5 | 15 |  |  | 4.95 | 4.34 | 7.0E-06 |
| Superior Temporal Gyrus / BA 38 | 45 | 7.5 | -24 | 5.1E-06 | 1802 | 5.88 | 4.96 | 3.5E-07 |
| Uncus / BA 28 | 34.5 | 3 | -25.5 |  |  | 5.62 | 4.80 | 8.0E-07 |
| * / Amygdala | 22.5 | -10.5 | -10.5 |  |  | 5.23 | 4.53 | 2.9E-06 |
| Cuneus / BA 17 | -12 | -85.5 | 6 | 3.6E-02 | 273 | 5.38 | 4.64 | 1.8E-06 |
| Insula / BA 13 | 40.5 | -21 | 7.5 | 1.0E-03 | 779 | 5.34 | 4.61 | 2.0E-06 |
| Superior Temporal Gyrus / BA 41 | 43.5 | -33 | 15 |  |  | 4.31 | 3.88 | 5.1E-05 |
| Uncus / BA 28 | -30 | 3 | -25.5 | 5.3E-03 | 527 | 5.21 | 4.53 | 3.0E-06 |
| Parahippocampal Gyrus / Hippocampus | -30 | -10.5 | -16.5 |  |  | 4.70 | 4.17 | 1.5E-05 |
| Superior Temporal Gyrus / BA 38 | -34.5 | 10.5 | -27 |  |  | 4.30 | 3.87 | 5.4E-05 |
| Insula / BA 13 | 33 | 21 | 4.5 | 3.8E-03 | 574 | 5.09 | 4.44 | 4.4E-06 |
| Claustrum / * | 30 | 9 | 7.5 |  |  | 4.23 | 3.82 | 6.6E-05 |
| Insula / BA 13 | -33 | 19.5 | 6 | 3.0E-02 | 294 | 4.96 | 4.35 | 6.8E-06 |
| Middle Frontal Gyrus / BA 8 | 27 | 21 | 45 | 3.2E-02 | 288 | 4.79 | 4.24 | 1.1E-05 |
| Medial Frontal Gyrus / BA 9 | 18 | 30 | 39 |  |  | 4.36 | 3.92 | 4.4E-05 |
| **GM; central brain details (local maxima more than 4mm apart)** | | | | | | | | |
| * / Amygdala | 22.5 | -10.5 | -10.5 | 8.5E-03 | 168 | 5.23 | 4.53 | 2.9E-06 |
| Parahippocampal Gyrus / BA 28 | -13.5 | -16.5 | -21 | 1.6E-02 | 110 | 5.18 | 4.50 | 3.4E-06 |
| Parahippocampal Gyrus / BA 35 | -18 | -21 | -15 |  |  | 4.68 | 4.16 | 1.6E-05 |
| Parahippocampal Gyrus / Amygdala | -30 | 1.5 | -24 | 1.7E-03 | 347 | 5.10 | 4.45 | 4.4E-06 |
| Parahippocampal Gyrus / Hippocampus | -30 | -10.5 | -16.5 |  |  | 4.70 | 4.17 | 1.5E-05 |
| **WHITE MATTER** | | | | | | | | |
| Cingulate Gyrus / * | -9 | -40.5 | 42 | 7.0E-03 | 738 | 5.65 | 4.82 | 7.2E-07 |
| Cingulate Gyrus / * | -7.5 | -31.5 | 39 |  |  | 4.88 | 4.29 | 8.8E-06 |
| Cingulate Gyrus / * | -7.5 | -31.5 | 30 |  |  | 4.31 | 3.88 | 5.2E-05 |
| Inferior Parietal Lobule / adjecent to BA2 | -48 | -28.5 | 33 | 1.4E-03 | 1114 | 5.07 | 4.43 | 4.7E-06 |
| Inferior Parietal Lobule / adjacent to BA 40 | -49.5 | -33 | 22.5 |  |  | 5.04 | 4.41 | 5.2E-06 |
| Inferior Parietal Lobule / adjacent to BA 40 | -57 | -34.5 | 30 |  |  | 4.56 | 4.07 | 2.4E-05 |
| Sub-Gyral / * | -21 | 28.5 | 31.5 | 9.4E-04 | 1220 | 5.03 | 4.40 | 5.5E-06 |
| Medial Frontal Gyrus / * | -12 | 42 | 27 |  |  | 4.97 | 4.36 | 6.4E-06 |
| Medial Frontal Gyrus / * | -18 | 54 | -3 |  |  | 4.49 | 4.02 | 2.9E-05 |
| Cingulate Gyrus / * | 13.5 | -33 | 42 |  |  | 4.13 | 3.75 | 9.0E-05 |
| Anterior Cingulate / * | 4.5 | 34.5 | 0 | 9.9E-03 | 663 | 4.36 | 3.92 | 4.4E-05 |
| Extra-Nuclear / * | 15 | 25.5 | 4.5 |  |  | 4.28 | 3.86 | 5.7E-05 |
| Extra-Nuclear / Corpus Callosum | 6 | 33 | 9 |  |  | 4.24 | 3.83 | 6.3E-05 |
| Posterior Cingulate / * | -10.5 | -54 | 10.5 | 5.3E-02 | 338 | 4.28 | 3.86 | 5.7E-05 |
| Posterior Cingulate / * | -13.5 | -54 | 18 |  |  | 4.21 | 3.81 | 7.1E-05 |
| Posterior Cingulate / adjacent to BA 29 | -4.5 | -48 | 15 |  |  | 4.14 | 3.76 | 8.6E-05 |
