# Supplementary material for: Cortical and Subcortical Grey and White Matter Atrophy in Myotonic Dystrophies Type 1 and 2 Is Associated with Cognitive Impairment, Depression and Daytime Sleepiness
Source: PLoS One. 2015 Jun 26;10(6):e0130352. doi: 10.1371/journal.pone.0130352 (PMC4482602; doi:10.1371/journal.pone.0130352)
Supplement: S3 Table — Areas of significant correlations between brain WM and depression score in DM (pooled data of DM1 and DM2) by voxelwise multiple regression analysis; areas with adjusted p at cluster level < 0.07 after FWE correction with local maxima more than 4 mm apart are shown; MNI coordinates: negative X-values reflect left side and positive X-values right sided location. (DOCX) [file pone.0130352.s003.docx]

**S3 Table: Depression and brain WM.**

Areas of significant correlations between brain WM and depression score in DM (pooled data of DM1 and DM2) by voxelwise multiple regression analysis; areas with adjusted p at cluster level < 0.07 after FWE correction with local maxima more than 4 mm apart are shown; MNI coordinates: negative X-values reflect left side and positive X-values right sided location.

| **WHITE MATTER** | | | | | | | | |
| --- | --- | --- | --- | --- | --- | --- | --- | --- |
| **Region** | **MNI coordinates** | | | **cluster-level** | | **peak-level** | | |
|  | **X** | **Y** | **Z** | **p (FWE-corr.)** | **equiv. cluster size (voxels)** | **T-score** | **equiv. Z-score** | **p (uncorr.)** |
| Pons / middle cerebellar peduncle | -16.5 | -27 | -33 | 5.47E-02 | 256 | 4.07 | 3.53 | 2.07E-04 |
| Pons / middle cerebellar peduncle | 21 | -31.5 | -39 | 6.93E-02 | 210 | 3.99 | 3.47 | 2.56E-04 |
| Pons / middle cerebellar peduncle | 13.5 | -39 | -46.5 |  |  | 3.70 | 3.27 | 5.30E-04 |
