# Supplementary material for: Cortical and Subcortical Grey and White Matter Atrophy in Myotonic Dystrophies Type 1 and 2 Is Associated with Cognitive Impairment, Depression and Daytime Sleepiness
Source: PLoS One. 2015 Jun 26;10(6):e0130352. doi: 10.1371/journal.pone.0130352 (PMC4482602; doi:10.1371/journal.pone.0130352)
Supplement: S4 Table — Areas of significant correlations between brain GM and WM and Epworth sleepiness score in DM2 by voxelwise multiple regression analysis with age as covariate; areas with adjusted p at cluster level < 0.07 after FWE correction with local maxima more than 8 mm apart are shown; MNI coordinates: negative X-values reflect left side and positive X-values right sided location. (DOCX) [file pone.0130352.s004.docx]

**S4 Table: ESS and brain GM and WM in DM2.**

Areas of significant correlations between brain GM and WM and Epworth sleepiness score in DM2 by voxelwise multiple regression analysis with age as covariate; areas with adjusted p at cluster level < 0.07 after FWE correction with local maxima more than 8 mm apart are shown; MNI coordinates: negative X-values reflect left side and positive X-values right sided location.

| **GREY MATTER** | | | | | | | | | |
| --- | --- | --- | --- | --- | --- | --- | --- | --- | --- |
| **Region** | **MNI coordinates** | | | | **cluster-level** | | **voxel-level** | | |
|  | **X** | **Y** | | **Z** | **equiv. cluster size (voxels)** | **p (FWE-corr.)** | **T-score** | **equiv. Z-score** | **p (uncorr.)** |
| **Medial Frontal Gyrus / BA 8** | 1.5 | 34.5 | | 48 | 551 | 3.9E-02 | 7.13 | 4.38 | 6.0E-06 |
| **Medial Frontal Gyrus / BA 8** | -3 | 30 | | 54 |  |  | 6.29 | 4.11 | 2.0E-05 |
| **Medial Frontal Gyrus / BA 8** | 3 | 24 | | 51 |  |  | 5.37 | 3.76 | 8.3E-05 |
| **WHITE MATTER** | | | | | | | | | |
| **Brainstem/ Midbrain** | 0 | -13.5 | -6 | | 499 | 6.9E-02 | 6.18 | 4.07 | 2.4E-05 |
| **Brainstem/ Midbrain** | -13.5 | -10.5 | -13.5 | |  |  | 5.25 | 3.72 | 1.0E-04 |
| **Middle cerebellar peduncle** | 28.5 | -55.5 | -36 | | 1114 | 3.3E-03 | 5.56 | 3.84 | 6.2E-05 |
| **Middle cerebellar peduncle** | -13.5 | -60 | -36 | | 493 | 7.0E-02 | 5.13 | 3.66 | 1.2E-04 |
| **Middle cerebellar peduncle** | -16.5 | -45 | -40.5 | |  |  | 4.13 | 3.20 | 7.0E-04 |
