# Supplementary material for: Cortical and Subcortical Grey and White Matter Atrophy in Myotonic Dystrophies Type 1 and 2 Is Associated with Cognitive Impairment, Depression and Daytime Sleepiness
Source: PLoS One. 2015 Jun 26;10(6):e0130352. doi: 10.1371/journal.pone.0130352 (PMC4482602; doi:10.1371/journal.pone.0130352)
Supplement: S5 Table — Areas of significant correlations between brain GM and flexibility of thinking (RWT test with change of categories) in DM1 and DM2 by voxelwise multiple regression analysis with type and age as covariates; areas with adjusted p at cluster level < 0.05 after FWE correction with local maxima more than 4 mm apart are shown; negative X-values reflect left side and positive X-values right sided location. (DOCX) [file pone.0130352.s005.docx]

**S5 Table: Flexibility of thinking and brain GM.**

Areas of significant correlations between brain GM and flexibility of thinking (RWT test with change of categories) in DM1 and DM2 by voxelwise multiple regression analysis with type and age as covariates; areas with adjusted p at cluster level < 0.05 after FWE correction with local maxima more than 4 mm apart are shown; negative X-values reflect left side and positive X-values right sided location.

| **GREY MATTER** | | | | | | | | |
| --- | --- | --- | --- | --- | --- | --- | --- | --- |
| **DM1** | | | | | | | | |
| **Region** | **MNI coordinates** | | | **cluster-level** | | **voxel-level** | | |
|  | **X** | **Y** | **Z** | **equiv. cluster size (voxels)** | **p (FWE-corr.)** | **T-score** | **equiv. Z-score** | **p (uncorr.)** |
| **Secondary visual cortex / BA 18** | -6 | -73.5 | -10.5 | 395 | 5.6E-03 | 8.95 | 4.44 | 4.5E-06 |
| **Secondary visual cortex / BA 18** | -9 | -78 | -13.5 |  |  | 8.70 | 4.39 | 5.6E-06 |
| **Secondary visual cortex / BA 19** | -6 | -69 | -4.5 |  |  | 8.45 | 4.34 | 7.1E-06 |
| **DM2** | | | | | | | | |
| **Periaqueductal Grey** | 1.5 | -36 | -7.5 | 1380 | 1.2E-04 | 7.03 | 4.35 | 6.9E-06 |
| **Midbrain/Red Nucleus** | -3 | -22.5 | -3 |  |  | 5.42 | 3.78 | 7.8E-05 |
| **Thalamus/Pulvinar** | 16.5 | -34.5 | 9 |  |  | 5.02 | 3.62 | 1.5E-04 |
| **Thalamus** | 6 | -24 | 12 |  |  | 5.00 | 3.61 | 1.6E-04 |
| **Midbrain/Red Nucleus** | -1.5 | -15 | -6 |  |  | 4.78 | 3.51 | 2.2E-04 |
| **Thalamus/Pulvinar** | -3 | -21 | 12 |  |  | 4.33 | 3.29 | 4.9E-04 |
| **Parahippocampal Gyrus / BA34** | -10.5 | -3 | -15 | 413 | 2.2E-02 | 8.14 | 4.66 | 1.6E-06 |
| **Anterior Cingulate / BA25** | -6 | -1.5 | -6 |  |  | 5.18 | 3.69 | 1.1E-04 |
| **Anterior Cingulate / BA25** | 7.5 | -6 | 1.5 |  |  | 4.50 | 3.38 | 3.6E-04 |
| **Anterior Cingulate / BA25** | 6 | 0 | -4.5 |  |  | 4.49 | 3.37 | 3.7E-04 |
| **Parahippocampal Gyrus / BA34** | 12 | -3 | -13.5 |  |  | 4.45 | 3.35 | 4.0E-04 |
