# Supplementary material for: Cortical and Subcortical Grey and White Matter Atrophy in Myotonic Dystrophies Type 1 and 2 Is Associated with Cognitive Impairment, Depression and Daytime Sleepiness
Source: PLoS One. 2015 Jun 26;10(6):e0130352. doi: 10.1371/journal.pone.0130352 (PMC4482602; doi:10.1371/journal.pone.0130352)
Supplement: S6 Table — ARWMC (age related white matter changes) score, the VRS (Virchow-Robin-spaces) score and the total FLAIR lesion load in DM1, DM2 and the healthy control group. Significance of group differences by Mann-Whitney-U tests (P > 0.050: not significant [n.s.], P < 0.050: significant, P <0.010: highly significant [bold]). (DOCX) [file pone.0130352.s006.docx]

**S6 Table: Lesion grading in DM1, DM2 and the healthy control group.**

ARWMC (age related white matter changes) score, the VRS (Virchow-Robin-spaces) score and the total FLAIR lesion load in DM1, DM2 and the healthy control group. Significance of group differences by Mann-Whitney-U tests (P > 0.050: not significant [n.s.], P < 0.050: significant, P <0.010: highly significant [bold]).

| **WM lesions**  **(ARWMC score)**  **mean+- SD** | **Controls**  **N=33** | **DM1**  **N=12** | **DM2**  **N=15** | **Significance**  **DM1 – controls** | **Significance**  **DM2 – controls** | **Significance**  **DM1 – DM2** |
| --- | --- | --- | --- | --- | --- | --- |
| **frontal** | **0.35**+-0.48 | **0.83**+-0.58 | **1.10**+-0.93 | P=0.049 | **P=0.004** | n.s. |
| **parieto-occipital** | **0.11**+-0.31 | **1.17**+-0.72 | **1.07**+-0.96 | **P<0.001** | **P<0.001** | n.s. |
| **temporal** | **-** | **0.33**+-0.65 | **0.47**+-0.81 | **P<0.001** | **P<0.001** | n.s. |
| **basal ganglia** | **-** | **0.08**+-0.29 | **0.07**+-0.26 | n.s. | n.s. | n.s. |
| **infra-tentorial** | **-** | **0.00** | **0.07**+-0.26 | n.s. | n.s. | n.s. |
| **VRS score** | ***** | **1.50**+-0.80 | **0.27**+-0.60 | n.s. | n.s. | **P<0.001** |
| **FLAIR lesion load / ml**  **median [range]** | **0.10**  **[0 – 1.8]** | **2.16**  **[0.12 – 10.9}** | **0.34**  **[0.01 - 98.6]** | **P<0.001** | P=0.013 | n.s. |

*widened VRS in basal ganglia found in 5 healthy controls

SD : standard deviation, ARWMC: age related white matter changes, VRS: Virchow-Robin spaces
